# Complete replacement of maize grain with sorghum and pearl millet grains in Jumbo quail diets: Feed intake, physiological parameters, and meat quality traits

**DOI:** 10.1371/journal.pone.0249371

**Published:** 2021-03-29

**Authors:** Thabiso Isaac Masenya, Victor Mlambo, Caven Mguvane Mnisi

**Affiliations:** 1 Department of Animal Science, School of Agricultural Science, North-West University, Mafikeng, South Africa; 2 School of Agricultural Sciences, Faculty of Agriculture and Natural Sciences, University of Mpumalanga, Nelspruit, South Africa; 3 Food Security and Safety Niche area, Faculty of Natural and Agricultural Science, North-West University, Mafikeng, South Africa; Tokat Gaziosmanpasa Universitesi, TURKEY

## Abstract

In sub-Saharan Africa, the use of maize (*Zea mays* L.) grain as an energy source in poultry feeds has become unsustainable due to competing demands and suboptimal growing conditions for the maize crop. Sorghum (*Sorghum bicolor* Moench L.) and pearl millet (*Pennisetum glaucum*) grains are potential sustainable alternatives, given their tolerance to local growing conditions. Therefore, this study evaluated the effect of total replacement of maize grain with whole or crushed sorghum and pearl millet grains on feed intake, and physiological and meat quality parameters of Jumbo quail. Five experimental diets were formulated by completely replacing crushed maize grain in a commercial grower diet (CON) with whole sorghum (WSG), crushed sorghum (CSG), whole millet (WMG), or crushed millet (CMG). Three hundred and fifty, two-week-old Jumbo quail chicks (74.7 ± 8.81 g live-weight) were evenly distributed into 35 replicate pens to which the experimental diets were allotted. Statistically similar (*P* > 0.05) weight gain and FCE values were observed between birds reared on the control and pearl millet-based diets. However, birds fed with sorghum-based diets had the lowest FCE and weight gain. Blood parameters fell within the normal ranges reported for healthy quail. Birds fed the whole sorghum grain diet had the least (*P* < 0.05) serum calcium and higher monocytes, cholesterol, and alkaline phosphatase (ALP) concentrations compared to those reared on the control diet. Compared to the control, the whole sorghum-containing diet reduced (*P* < 0.05) carcass, breast, wing, thigh, drumstick, liver, gizzard, and large intestine weights of the birds. Complete replacement of maize grain with pearl millet grain (whole or crushed) did not compromise feed intake, growth performance, and meat quality traits of the Jumbo quail birds. However, whole sorghum grain reduced growth performance of the birds.

## Introduction

A sustainable increase in poultry production could significantly contribute to the dietary needs of a growing human population and thus guarantee food and nutrition security. The Jumbo quail (*Coturnix* sp.) has emerged as an important poultry species that is reared intensively for meat production in South Africa. According to Mbhele et al. [1], the Jumbo quail is a larger version of the Japanese quail (*Coturnix coturnix japonica*) that has been recently developed for meat production. This bird weighs up to an average of 250 g at six weeks of age [2] compared to a six-week old Japanese quail that weighs an average of 160 g [3]. Commercial quail diets still rely heavily on maize (*Zea mays* L.) grain as the major energy source [4], which poses a major challenge to quail farmers. This is because maize grain is also a staple food for humans in sub-Saharan Africa, resulting in high market prices for this commodity [3]. Apart from being a major dietary ingredient for farmed animals, maize grain is also used in the production of various beverages, biofuels, commercial starch, and human food [5]. Furthermore, production of high-yielding maize varieties is a capital-intensive pursuit [4], characterized by high usage of synthetic fertilizers and irrigation water. It is, therefore, imperative that alternative dietary energy sources be identified and evaluated as a contribution to sustainable quail farming.

Small grains such as sorghum (*Sorghum bicolor* Moench L.) and pearl millet (*Pennisetum glaucum L*.), are potential dietary energy sources that could be used to replace maize in Jumbo quail diets. Sorghum and millet are drought tolerant plants that produce nutrient-rich grains [6, 7] whose feed value for the Jumbo quail is largely unknown. This is despite the existence of several studies where these small grains have been evaluated in other avian species [7–9]. Low-tannin sorghum grain has been reported to be a suitable alternative to maize grain in some poultry diets [9]. However, the presence of kafirin, endosperm cell wall and ferulic acid, tannins, and phytate in sorghum grain limits its utilization as an energy source [10]. This could be the reason why discordant results have been reported when sorghum grain has been used in place of maize grain in poultry diets. Pearl millet grain contains higher protein content, but lower energy compared to maize grain [11]. While Cisse et al. [6] report that pearl millet grain is an effective alternative energy source in layers and broiler chickens, results of trials where maize grain was replaced with pearl millet grain have been inconsistent [6, 7, 12].

An important question when using small grains in poultry diets is whether they should be fed whole or crushed. Liu et al. [8] suggest that particle size reduction in white sorghum grain improves nutrient utilization and growth performance in chickens. However, Selle et al. [12] found no nutritional benefit of reducing particle size in red sorghum grains. Reducing particle size is a costly and laborious exercise for farmers and feed manufacturers [13], so it is important to investigate whether this practice is essential. The utility of sorghum and pearl millet grains as dietary energy sources has not been evaluated in Jumbo quail until now. Therefore, this study investigated the effect of complete replacement of maize grain with whole or crushed sorghum and pearl millet grains on feed intake, growth performance, blood parameters, carcass characteristics, internal organs, and meat quality traits of Jumbo quail. We hypothesized that diets containing whole or crushed sorghum and pearl millet grains would promote similar feed intake, physiological parameters, and meat quality traits in Jumbo quail as the maize grain-based control diet.

## Materials and methods

### Research area and resources

The study was conducted in summer at Molelwane Research Farm (25°40.459′ S; 26°10.563′ E; Altitude: 1225 m above sea level) of the North-West University North-West, South Africa). Ambient temperatures around this area ranges between 22°C and 37°C, with an average annual rainfall of 450 mm [3]. White sorghum and pearl millet grains were purchased from Alzu Depots Midrand SA PTY (LTD) (Gauteng, South Africa). Other feed ingredients were acquired from Simplegrow Agric Services PTY (LTD) (Gauteng, South Africa).

### Diet formulation and analyses

Five dietary treatments (Table 1) were formulated to meet the requirements for quail birds as recommended by the National Research Council [14]. The diets were formulated by total replacement of crushed maize grain in a commercial grower diet (CON) with whole sorghum (WSG), crushed sorghum (CSG), whole millet (WMG), or crushed millet (CMG).

**Table 1:** Ingredient and chemical composition (g/kg as fed basis, unless stated otherwise) of experimental diets.

	^1^Diets				
Ingredients	CON	WSG	CSG	WMG	CMG
Maize grain	579.2	0	0	0	0
Sorghum grain	0	579.2	579.2	0	0
Pearl millet grain	0	0	0	579.2	579.2
Soybean meal	339.2	339.2	339.2	339.2	339.2
Sunflower oil	45.0	45.0	45.0	45.0	45.0
Limestone	10.9	10.9	10.9	10.9	10.9
Dicalcium phosphate	16.9	16.9	16.9	16.9	16.9
Salt (fine)	3.3	3.3	3.3	3.3	3.3
Sodium bicarbonate	0.01	0.01	0.01	0.01	0.01
Vitamin + mineral premix^2^	2.0	2.0	2.0	2.0	2.0
DL-Methionine	1.6	1.6	1.6	1.6	1.6
L-Lysine	1.8	1.8	1.8	1.8	1.8
**Chemical composition**					
Dry matter	908.3	908.6	908.6	913.5	913.5
Crude protein	161.9	168.3	168.3	186.7	186.7
Metabolisable energy (kcal/kg)	2572.1	2539.7	2539.7	2525.2	2525.2
Ash	62.6	55.1	55.1	59.5	59.5
Organic matter	845.7	853.4	853.4	854.0	854.0
Crude fibre	31.2	36.1	36.1	38.3	38.3
Starch	335.7	413.2	413.2	408.9	408.9

^1^Diets: CON = maize grain-based commercial grower diet; WSG = commercial grower diet in which maize grain was replaced with whole sorghum grain; CSG = commercial grower diet in which maize grain was replaced with crushed sorghum grain; WMG = commercial grower diet in which maize grain was replaced with whole millet grain; CMG = commercial grower diet in which maize grain was replaced with crushed millet grain.

^2^Premix: vitamin A (11000 IU), vitamin D3 (2500 IU), vitamin E (25 IU), vitamin K3 (2.0 mg), vitamin B1 (2.5 mg), vitamin B2 (4.5 mg), vitamin B6 (5.1 mg), niacin (30 mg), pantothenic acid (10 mg), folic acid (0.7 mg), biotin (0.12 g), copper sulphate (8.0 mg), potassium iodide (0.34 mg), ferrous sulphate (80 mg), magnesium sulphate (100 mg), sodium selenite (0.25 mg), and zinc sulphate (79 mg).

Proximate constituents (dry matter, crude protein, ash, organic matter, crude fibre, and starch) of the diets were determined using the Association of Official Analytical Chemists methods [15]. The metabolisable energy (ME) content of the diets was calculated using the following formula: *ME* (*kcal/kg*) = 2778–66 (*Crude Fibre* × 88% *DM*) as described by Livesey [16].

### Animal rights statement and feeding trial

The research methodologies as well as the feeding and slaughtering procedures employed in this study were evaluated and approved (approval no: NWU-01888-19-S5) by the Animal Production Research Ethics Committee of the North-West University (North West, South Africa). Three hundred and fifty, one-week-old unsexed Jumbo quail chicks were purchased from T/A R&G Poultry in Walkerville (Gauteng, South Africa). The chicks were then randomly and evenly allocated to 35 pens (experimental units), such that each dietary treatment was replicated seven times. The birds were then acclimatized to the five experimental diets and their pens for a week before measurements commenced [1]. Each replicate pen (100 cm long × 60 cm wide × 30 cm high) carried 10 birds. The pens were constructed using wire-mesh with slatted steel floors that require no bedding. The birds had free access to feed and water, which were offered using oval hole feed tubes that minimize waste and poultry drinkers (Poltek Poultry Water Fountain, Mimosa Chicks, South Africa) respectively. Birds were offered feed *ad libitum* every morning after the collection and weighing of feed refusals. These data were used to calculate average weekly feed intake (AWFI) as shown in S1 Table. At two weeks of age, all the birds in each pen were weighed (Explorer EX224, OHAUS Corp, New Jersey, USA) to measure the initial live-weights (74.7 ± 8.81 g live-weight) and subsequently weighed weekly (S2 Table) until the age of six weeks to determine average weekly body weight gain (ABWG). Feed conversion efficiency (FCE) was calculated by dividing body weight gain with feed intake. The feeding trial was conducted under natural lighting.

### Slaughter procedure and blood analyses

At 6 weeks of age, all the birds were electrically stunned and slaughtered by cutting the jugular vein using a sharp knife in a locally registered poultry abattoir (North West, South Africa). While bleeding, 2 mL of blood samples were collected from each of two randomly selected birds per replicate pen into hematological and serum biochemical tubes. The hematological tubes contained an anti-coagulant ethylenediaminetetraacetic acid (EDTA) while no anticoagulant was used for the serum biochemistry. The clotted blood was centrifuged in a centrifuge (Hermle Labortechnik GmbH, Germany) at 1000 *g* for 15 min to generate the serum [17]. Hematological and serum biochemical indices were analyzed using an automated IDEXX LaserCyte Hematology and an automated IDEXX Vet Test Chemistry Analyzers from IDEXX Laboratories (PTY) LTD (Gauteng, South Africa) following the guidelines by Washington & van Hoosier [17].

### Carcass traits and internal organs

After plucking and manual evisceration, the carcasses (excluding the head, necks, and feet) were measured to determine hot carcass and cold carcass weights. Weights of internal organs (liver, gizzard, proventriculus, and small and large intestines) and carcass cuts (breast, wing, drumstick, and thigh) were measured for every experimental unit.

### Meat quality measurements

Breast meat color coordinates (L* = luminosity; a* = red color intensity; and b* = yellow color intensity) were determined 24 h post-mortem using a spectrophotometer (CM 2500c model, Konica Minolta Inc., Japan) following the Commission Internationale de l’Eclairage guidelines [18]. A portable digital pH meter (CRISON pH25, CRISON Instruments SA, Spain) with a piercing electrode was used to measure the pH in breast muscles 24 h post-mortem. The pH meter was calibrated after measuring each replicate treatment as prescribed by the manufacturer. The pressure method was used to determine the water holding capacity (WHC) as described by Grau & Hamm [19]. Thaw loss measurement was determined using a method adapted from Zhang et al. [20]. For cooking loss, raw breast muscle samples were individually weighed and oven-broiled until the samples reached an internal temperature of 75°C as described by Honikel [21]. Thereafter, breast meat samples from each experimental unit were sheared using a Meullenet-Owens Razor Shear Blade mounted on a Texture Analyzer (TA-XT plus, Stable Micro Systems, Surrey, UK) to determine shear force values (N).

### Data analysis

Weekly measured data were analyzed using the repeated measures analysis in the general linear model procedure of the Statistical Analysis System (SAS) version 9.4 [22], where the following statistical linear model was employed:

$$Y_{ijk}=\mu +D_{i}+W_{j}+{\left(D\times W\right)}_{ij}+E_{ijk}$$

Where, *Y*_*ijk*_ = dependant variable, *μ* = overall population mean, *D*_*i*_ = effect of diet, *W*_*j*_ = effect of time, *(D × W)*_*ij*_ = interaction effect of diet and time, and *E*_*ijk*_ = random error term associated with observation *ijk*, assumed to be normally and independently distributed.

Data for overall feed intake, weight gain, FCE, blood indices, internal organ weights, carcass characteristics and meat quality were analyzed using the following general linear model procedure of SAS version 9.4 [22]:

$$Y_{ij}=\mu +D_{i}+E_{ij}$$

Where, *Y*_*ij*_ = the dependent variable, *μ* = overall population mean, *D*_*i*_ = dietary effect, and *E*_*ij*_ = random error term associated with observation *ij*, assumed to be normally and independently distributed. The level of significance was set at *P* < 0.05, and the probability of difference option in SAS was used to separate the least square means. The Bonferroni adjustment of the significance level (α) was used for multiple comparisons.

## Results

### Feed intake and growth performance

Repeated measures analysis showed significant week × diet interaction effects on ABWG, FCE, but not on AWFI (*P* = 0.291). Quail birds reared on crushed sorghum grain-containing diet had the highest overall feed intake (3093.3 g/bird) while the lowest intake was observed in birds reared on whole sorghum grain-containing diet (2940.8 g/bird). Nonetheless, overall feed intake was similar (*P* > 0.05) in birds offered diets CON, WMG, CSG, and CMG.

Table 2 shows that the sorghum grain-containing diets (WSG and CSG) promoted lower (*P* = 0.0001) weight gains in three-week old birds compared to the rest of the diets. In week 4, the whole sorghum grain-containing diet (41.50 g/bird) promoted lower (*P* = 0.001) weight gain than the other diets. Five-week-old birds reared on the whole sorghum grain-containing diet had the least weight gain (99.08 g/bird) compared to all the other diets. However, the sorghum grain-containing diets (WSG and CSG) promoted similar weight gain. No dietary effects (*P* = 0.916) were observed for weight gain in week 6.

**Table 2:** Average weekly body weight gain (g/bird) in Jumbo quail reared on whole or crushed sorghum and pearl millet grains-based diets.

	^1^Diets						
	CON	WSG	CSG	WMG	CMG	^2^SEM	*P* value
Week 3	52.35^b^	32.62^a^	40.59^a^	56.73^b^	55.63^b^	2.031	0.0001
Week 4	91.67^b^	41.50^a^	80.05^b^	91.98^b^	84.60^b^	5.455	0.001
Week 5	123.8^b^	99.08^a^	112.5^ab^	127.0^b^	124.2^b^	4.399	0.0001
Week 6	142.4	149.3	135.3	145.7	140.4	12.11	0.916

^a,b^ In row, means with common superscripts do not differ (*P* > 0.05).

^1^Diets: CON = maize grain-based commercial grower diet; WSG = commercial grower diet in which maize grain was replaced with whole sorghum grain; CSG = commercial grower diet in which maize grain was replaced with crushed sorghum grain; WMG = commercial grower diet in which maize grain was replaced with whole millet grain; CMG = commercial grower diet in which maize grain was replaced with crushed millet grain.

^2^SEM = standard error of the mean.

Three-week old birds fed sorghum grain-containing diets (WSG and CSG) had lower FCE than those fed with the other diets, which were statistically similar (Table 3). In week 4, the whole sorghum grain-containing diet promoted the least FCE (0.051 g:g) in quail birds compared to all the other diets. Five-week-old birds fed the whole sorghum grain-containing diet had the least FCE (0.153 g:g), however, birds fed sorghum grain-containing diets (WSG and CSG) had similar (*P =* 0.088) FCE. In week 6, similar (*P* = 0.545) FCE was recorded in the birds across all the diets.

**Table 3:** Average weekly feed conversion efficiency (g:g) in Jumbo quail reared on whole or crushed sorghum and pearl millet grains-based diets.

	^1^Diets						
	CON	WSG	CSG	WMG	CMG	^2^SEM	*P* value
Week 3	0.094^b^	0.060^a^	0.074^a^	0.103^b^	0.101^b^	0.004	<0.0001
Week 4	0.104^b^	0.051^a^	0.091^b^	0.107^b^	0.096^b^	0.006	0.002
Week 5	0.189^b^	0.153^a^	0.174^ab^	0.194^b^	0.190^b^	0.007	<0.0001
Week 6	0.146	0.161	0.133	0.145	0.145	0.013	0.545

^a,b^ In row, means with common superscripts do not differ (*P* > 0.05).

^1^Diets: CON = maize grain-based commercial grower diet; WSG = commercial grower diet in which maize grain was replaced with whole sorghum grain; CSG = commercial grower diet in which maize grain was replaced with crushed sorghum grain; WMG = commercial grower diet in which maize grain was replaced with whole millet grain; CMG = commercial grower diet in which maize grain was replaced with crushed millet grain.

^2^SEM = standard error of the mean.

### Hematological and serum biochemical parameters

Table 4 shows that there were no significant dietary effects (*P* > 0.05) observed on all hematological parameters except for monocytes (P = 0.017). The whole sorghum grain-containing diet promoted higher monocytes (68.02%) compared to the control, crushed sorghum and whole millet grain-containing diets. Similar (*P* = 0.108) levels of monocytes were also recorded in birds offered whole sorghum and crushed millet grain-containing diets.

**Table 4:** Hematological parameters in Jumbo quail reared on whole or crushed sorghum and pearl millet grains-based diets.

	^1^Diets						
^2^Parameters	CON	WSG	CSG	WMG	CMG	^3^SEM	*P* value
Eosinophils (×10^9^/L)	2.38	3.50	2.20	2.25	3.13	0.554	0.337
Erythrocytes (×10^12^/L)	1.73	1.42	1.46	1.63	1.48	0.233	0.832
Hemoglobin (g/dL)	8.75	9.28	9.38	9.05	10.37	1.086	0.822
Lymphocytes (×10^9^/L)	70.83	123.8	66.04	81.85	108.1	22.60	0.304
MCH (pg)	55.27	57.74	60.03	51.74	50.94	10.85	0.963
MCV (fL)	25.18	21.28	26.90	22.44	24.74	2.872	0.504
Monocytes (×10^9^/L)	32.80^a^	68.02^b^	30.46^a^	32.40^a^	48.82^ab^	7.735	0.017
MPV (fL)	2.20	2.24	2.33	2.37	2.28	0.091	0.563
Neutrophils (×10^9^/L)	19.81	50.62	18.72	24.70	24.08	8.612	0.082
PDW (%)	12.28	11.94	12.18	13.07	12.32	0.395	0.297
Platelets (K/μL)	2260	2500	1869	2500	2189	219.9	0.244
RDW (×10^9^/L)	32.33	29.98	37.10	8.98	34.74	7.769	0.865
Reticulocytes (K/μL)	0.42	0.04	0.46	0.40	0.22	0.303	0.676
White blood cell (×10^9^/L)	162.7	211.0	123.1	141.2	176.8	29.48	0.282

^a,b^ In row, means with common superscripts do not differ (*P* > 0.05).

^1^Diets: CON = maize grain-based commercial grower diet; WSG = commercial grower diet in which maize grain was replaced with whole sorghum grain; CSG = commercial grower diet in which maize grain was replaced with crushed sorghum grain; WMG = commercial grower diet in which maize grain was replaced with whole millet grain; CMG = commercial grower diet in which maize grain was replaced with crushed millet grain.

^2^Parameters: MCV = mean corpuscular volume; MCH = mean corpuscular hemoglobin; MPV = mean platelet volume; PDW = platelet distribution width; RDW = red blood cell distribution width.

^3^SEM = standard error of the mean.

Table 5 shows that the dietary treatments only affected (*P* < 0.05) serum calcium, cholesterol, amylase, and alkaline phosphatase (ALP) levels. Birds fed the whole sorghum grain-containing diet had lower (*P* = 0.002) serum calcium (2.24 mmol/L) compared to all the other diets. The whole sorghum grain-containing diet (5.07 mmol/L) promoted higher cholesterol levels compared to the whole millet grain diet (3.13 mmol/L). However, cholesterol levels of the birds fed with all the other diets (CON, CSG, WMG and CMG) were statistically similar. Quail birds fed the diet containing crushed millet grain had higher serum amylase (457.5 U/L) than those fed with whole sorghum grain-containing diet (278.5 U/L). Quail birds reared on the whole sorghum grain diets had higher ALP (457.5 U/L) than those fed with the other diets, whose ALP levels did not differ.

**Table 5:** Serum biochemical parameters in Jumbo quail reared on whole or crushed sorghum and pearl millet grains-based diets.

	^1^Diets						
^2^Parameters	CON	WSG	CSG	WMG	CMG	^3^SEM	*P* value
ALB/GLOB	0.81	0.50	0.57	1.49	0.59	0.439	0.503
Albumin (g/L)	18.36	16.79	17.07	18.57	22.93	2.377	0.386
ALP (U/L)	128.5^a^	457.5^b^	151.4^a^	174.4^a^	84.29^a^	42.35	<0.0001
ALT (U/L)	67.46	66.14	67.50	64.57	61.0	7.809	0.974
Amylase (U/L)	338.1^ab^	278.5^a^	435.4^ab^	294.8^ab^	457.5^b^	43.77	0.019
Bilirubin (μmol/L)	37.36	22.5	24.64	19.86	27.03	5.200	0.181
Calcium (mmol/L)	3.60^b^	2.24^a^	3.54^b^	3.49^b^	3.33^b^	0.237	0.002
Cholesterol (mmol/L)	3.73^a^	5.07^b^	4.66^ab^	3.13^ab^	3.54^ab^	0.447	0.024
GGT (U/L)	1.00	1.29	0.43	0.57	0.50	0.696	0.887
Globulin (g/L)	37.36	34.86	34.50	34.0	29.09	2.600	0.276
Glucose (mmol/L)	1.57	1.01	1.81	1.17	1.87	0.284	0.153
Lipase (U/L)	333.9	437.4	277.2	349.9	326.9	40.60	0.110
SDMA (μg/dL)	22.36	25.79	26.27	21.43	21.29	1.941	0.213
Total protein (g/L)	52.83	50.86	51.57	48.43	57.0	4.633	0.762
Urea (mmol/L)	1.06	1.09	1.07	1.20	1.20	0.076	0.493

^a,b^ In row, means with common superscripts do not differ (*P* > 0.05).

^1^Diets: CON = maize grain-based commercial grower diet; WSG = commercial grower diet in which maize grain was replaced with whole sorghum grain; CSG = commercial grower diet in which maize grain was replaced with crushed sorghum grain; WMG = commercial grower diet in which maize grain was replaced with whole millet grain; CMG = commercial grower diet in which maize grain was replaced with crushed millet grain.

^2^Parameters: ALB*/*GLOB = albumin-to-globulin ratio; ALP = alkaline phosphatase; ALT = alanine aminotransferase; GGT = gamma-glutamyl transferase; SDMA = symmetric dimethylargininase.

^3^SEM = standard error of the mean.

### Carcass traits, internal organ weights and meat quality

Table 6 shows that there were significant dietary effects on all carcass characteristics and internal organs except for proventriculus and small intestine (*P* > 0.05). Quail fed with whole sorghum grain-based diet had lower (*P* < 0.001) hot carcass, cold carcass, breast, and thigh weights compared to those fed with the other diets. Birds reared on the control group had statistically similar wing weights as those reared on the other diets. Birds fed the diet containing whole millet grain had the heaviest drumstick weights (7.42 g/bird) while those fed the diet containing whole sorghum grain had the lightest drumstick weights (5.53 g/bird). Birds reared on the control group had similar (*P* > 0.05) drumstick weights as those reared on crushed sorghum- and millet-based groups. Birds reared on the whole sorghum group promoted lower liver weights (3.44 g/bird) than those reared on the control, crushed sorghum and whole millet groups. Birds fed the crushed sorghum grain-based diet had the heaviest gizzards (4.77 g/bird) while those fed the whole sorghum grain-based diet had the lightest gizzards (2.87 g/bird). Birds fed the control diet had statistically similar gizzard weight as those fed the millet grain-based diets. Birds fed the whole millet grain-based diet had heavier large intestines (2.65 g/bird) than those fed the whole sorghum grain-based diet (1.82 g/bird), while birds fed the control and crushed millet or sorghum grain-based (CSG and CMG) diets had statistically similar large intestine weights.

**Table 6:** Carcass characteristics and internal organ sizes (g/bird) in Jumbo quail reared on whole or crushed sorghum and pearl millet grains-based diets.

	^1^Diets						
Parameters	CON	WSG	CSG	WMG	CMG	^2^SEM	*P* value
Warm carcass	157.37^b^	110.96^a^	146.19^b^	157.18^b^	152.70^b^	5.346	<0.0001
Cold carcass	150.89^b^	107.56^a^	142.12^b^	153.13^b^	146.62^b^	4.908	<0.0001
Breast	37.96^b^	23.47^a^	34.1^b^	38.48^b^	36.10^b^	2.471	0.001
Wing	7.16^ab^	6.14^a^	6.68^ab^	7.35^b^	7.0^ab^	0.256	0.020
Drumstick	7.03^bc^	5.53^a^	6.55^b^	7.42^c^	6.98^bc^	0.193	<0.0001
Thigh	10.51^b^	6.56^a^	9.85^b^	10.85^b^	10.39^b^	0.408	<0.0001
Liver	4.42^b^	3.44^a^	4.64^b^	4.65^b^	4.27^ab^	0.231	0.016
Gizzard	3.86^b^	2.87^a^	4.77^c^	3.66^b^	3.72^b^	0.129	<0.001
Proventriculus	0.96	0.90	1.08	0.98	0.94	0.176	0.949
Small intestine	6.47	5.36	6.84	6.71	6.64	0.389	0.067
Large intestine	2.11^ab^	1.82^a^	1.93^ab^	2.65^b^	2.24^ab^	0.208	0.078

^a,b,c^ In row, means with common superscripts do not differ (*P* > 0.05).

^1^Diets: CON = maize grain-based commercial grower diet; WSG = commercial grower diet in which maize grain was replaced with whole sorghum grain; CSG = commercial grower diet in which maize grain was replaced with crushed sorghum grain; WMG = commercial grower diet in which maize grain was replaced with whole millet grain; CMG = commercial grower diet in which maize grain was replaced with crushed millet grain.

^2^SEM = standard error of the mean.

Diets had no effect (*P* > 0.05) on all meat quality parameters except for meat pH (Table 7). Meat from birds reared on whole sorghum group had higher pH (6.20) compared to meat from the crushed millet group (5.73). Meat from birds reared on the control group had statistically similar pH as meat from those reared on all the other diets.

**Table 7:** Meat quality parameters in Jumbo quail reared on whole or crushed sorghum and pearl millet grains-based diets.

	^1^Diets						
Parameters	CON	WSG	CSG	WMG	CMG	^3^SEM	*P* value
pH	5.87^ab^	6.20^b^	5.80^ab^	5.81^ab^	5.73^a^	0.057	0.0001
Lightness (L*)	49.04	48.29	48.30	49.10	49.83	0.499	0.186
Redness (a*)	6.38	5.11	4.84	18.30	6.89	5.121	0.324
Yellowness (b*)	9.85	9.58	9.40	8.72	9.11	0.382	0.292
^2^WHC (%)	10.67	15.22	14.26	13.57	12.18	2.135	0.597
Thaw loss (%)	16.26	21.02	15.38	19.40	20.82	3.217	0.624
Cooking loss (%)	44.25	45.02	39.30	36.47	40.90	2.984	0.256
Shear force (N)	2.12	1.93	2.14	2.18	2.11	0.076	0.188

^a,b^ In row, means with common superscripts do not differ (*P* > 0.05).

^1^Diets: CON = maize grain-based commercial grower diet; WSG = commercial grower diet in which maize grain was replaced with whole sorghum grain; CSG = commercial grower diet in which maize grain was replaced with crushed sorghum grain; WMG = commercial grower diet in which maize grain was replaced with whole millet grain; CMG = commercial grower diet in which maize grain was replaced with crushed millet grain.

^2^WHC = water holding capacity.

^3^SEM = standard error of the mean.

## Discussion

### Feed intake and growth performance

Several studies have been conducted to determine the effect of feeding sorghum and millet grains as major energy sources in poultry nutrition [6, 9]. However, no studies have investigated the effect of replacing maize with whole or crushed sorghum and millet grains in Jumbo quail diets. Results from the current study showed that replacing maize grain with whole sorghum grain resulted in poor overall feed intake and growth performance from weeks 3–5 of age. This indicates that the Jumbo quail had difficulties consuming and efficiently utilizing whole sorghum grains. In contrast, using crushed sorghum grain as an energy source improved feed intake and growth performance than when whole sorghum grain was used in this study. These results corroborate the findings by Ahmed et al. [23], where Ross broilers performed poorly when reared on diets in which maize grain had been completely replaced with sorghum grains. Indeed, Leandro et al. [24] reported that smaller feed particles improve nutrient digestibility, feed utilization, and growth performance in quail. In this study, replacing maize grain with pearl millet grain resulted in similar body weight gain and FCE suggesting that this small grain is a suitable alternative to maize grain even when fed as a whole grain. Lower weight gain in birds fed the sorghum grain-containing diets (WSG and CSG) in this study was consistent with the findings of Gualtieri & Rapaccini [25], who reported poorer weight gains in broilers offered sorghum-based diets. The utilization of sorghum grain as an energy source in birds can be limited by the presence of kafirin [10], tannins, endosperm cell wall [26], ferulic acid [27] and phytate [28, 29]. Indeed, kafirin, the dominant protein in sorghum grain, is poorly digested in birds due to its hydrophobicity [30]. Consequently, the digestibility of sorghum starch has been reported to be 7.05% lower than that of maize starch due to the close physical proximity of starch granules to kafirin protein granules [30]. This could explain the poor performance of the birds reared on sorghum grain-based diets in this study.

### Hematological and serum biochemical parameters

Blood indices reflect the pathophysiological response of the bird to its environmental factors and provide an opportunity for clinical investigation [31]. In this study, blood parameters were used to assess the safety and quality of experimental diets in relation to the control. For hematological parameters, diets only influenced the concentration of monocytes in quail birds. Birds fed the whole sorghum grain-based diet exhibited the highest monocytes level in contrast with a previous study [32], where a decrease in monocytes in birds fed sorghum (*Tumbuna* cultivars)-based diets compared to those fed maize-grain based diets was reported. In addition, the blood parameters fell within the normal ranges reported for healthy quail birds [1, 3].

Regarding serum biochemical indices, diets only affected calcium, cholesterol, amylase, and ALP levels while no differences were observed for all the other biomolecules. The whole sorghum grain-based diet reduced the calcium and amylase concentrations, which could be due to poor intake and utilization of the sorghum-based diet. The enzyme, ALP, was highest for birds reared on the whole sorghum diet, indicating possible malnutrition [2]. This shows that feeding whole sorghum grains to quail has adverse effects on their energy and mineral nutrition. In contrast, serum amylase levels were higher in quail fed maize, crushed sorghum and millet (crushed and whole millet) grains, which could be a response to high starch intake.

### Carcass traits, internal organ weights and meat quality

Birds offered the whole sorghum grain-based diet had lower carcass weights and cuts (breast, wing, drumstick, and thigh weights), which was consistent with the findings of Tandiang et al. [33] who reported that substituting maize corn with low-tannin sorghum grain resulted in lower weight gains in broiler chickens. Feeding whole sorghum-based diets may reduce profitability of quail farming because carcass traits are used to determine market prices in the commercial world. On the other hand, crushed sorghum grain-based diet promoted similar carcass traits as the maize and millet grains-containing diets, suggesting the need to crush whole sorghum grains before inclusion in quail diets. Efficient feed utilization is important in encouraging muscle gain and fat deposition, thus the reduced carcass values in the whole sorghum group can be attributed to the low intake and utilization of whole sorghum grain nutrients.

Diets affected the weights of all internal organs except for proventriculi and small intestines. According to Xu et al. [34], rearing broiler chickens on larger feed particles such as whole sorghum grain-containing diets has several potential benefits that include increased enzymatic digestion and stimulating gizzard muscle development. However, the opposite effect was observed in this study, where feeding whole sorghum grain reduced the gizzard and liver weights of the Jumbo. These results also contradict those of Issa et al. [35], who reported no changes in gizzard and liver weights in broilers when maize grain was substituted with sorghum grains. In other studies, replacing maize with pearl millet grain had no significant effect on gizzard weight and size of small and large intestines [36], giblets weights, liver, gizzard, and abdominal fat [37] of broiler chickens. Higher large intestine weights were expected in the whole sorghum group as an adaptation mechanism to larger feed particles. However, the depressed intake of the whole sorghum grain-based diet could explain why the birds’ large intestine weights were smaller than those reared on the whole millet group whose intake was higher.

Total replacement of maize grain with whole or crushed sorghum and millet grains did not affect meat quality parameters of the Jumbo quail, except for breast meat pH, which was highest in the whole sorghum group (6.20) and lowest in the crushed millet group (5.73). Meat pH is a trait reported to have greater influence on several meat quality traits [38]. For example, several authors reported a significant correlation between post-mortem breast meat pH values and color values, where a lower pH value results in lighter or pale meat, while a higher pH value causes dark meat color [38, 39]. The rate at which post-mortem pH declines is influenced by the conversion of glycogen levels in the muscle into lactic acid [38], thus the higher ultimate pH value (6.20) observed in the whole sorghum group could be an indication of low glycogen levels in the quail birds prior to slaughter. Indeed, excessive starving or malnutrition have been reported to cause high post-mortem pH values in animals [40]. It is not surprising that meat from whole sorghum birds may have had low glycogen levels given the low feed intake and poor growth performance. Low pH values (<5.4) in poultry meat have been associated with reduced water-holding capacity, which in turn results in increased thawing and cooking losses [41]. However, none of these parameters were affected by the change in breast meat pH. Replacing maize grain with whole or crushed millet and sorghum grain promoted similar meat lightness values. Lightness is a good indicator of the freshness of meat and has a direct influence on the final purchase decision of consumers [42].

## Conclusions

Total replacement of crushed maize grain with whole or crushed pearl millet grains did not cause adverse effects on growth performance, hematological and serum biochemical indices, size of internal organs, carcass characteristics, and meat quality parameters. However, whole sorghum grain compromised physiological and meat quality parameters of the birds and should be crushed prior inclusion in quail diets. Although pearl millet grain is a suitable maize grain alternative in quail diets, a cost-benefit analysis may be necessary since pearl millet grain is currently more expensive than maize grain.

## Supporting information

S1 Table Average weekly feed intake (g/bird) in Jumbo quail reared on whole or crushed sorghum and pearl millet grains-based diets. (DOCX)

S2 Table Average weekly body weight (g/bird) in Jumbo quail reared on whole or crushed sorghum and pearl millet grains-based diets. (DOCX)

## References

[1] Mbhele FGT, Mnisi CM, Mlambo V. A nutritional evaluation of insect meal as a Sustainable protein source for Jumbo quails: Physiological and meat quality responses. Sustainability. 2019; 11:6592.

[2] Marareni M, Mnisi CM. Growth performance, serum biochemistry and meat quality traits of Jumbo quails fed with mopane worm (*Imbrasia belina*) meal-containing diets. Veterinary and Animal Science. 2020; 10:100141. 10.1016/j.vas.2020.100141 32923738 PMC7475268

[3] Mnisi CM, Mlambo V. Growth performance, hematology, and serum biochemistry and meat quality characteristics of Japanese quail (*Coturnix coturnix japonica*) fed canola meal-based diets. Animal Nutrition. 2018; 4:37–43. 10.1016/j.aninu.2017.08.011 30167482 PMC6112353

[4] Morne P. Facts and Trends SA: Agriculture. Production vs Demand, South Africa, Pretoria. 2017. p. 1–3.

[5] Tenenbaum DJ. Food vs. fuel: diversion of crops could cause more hunger. Environmental Health Perspectives. 2008; 116:254–257. 10.1289/ehp.116-a254 18560500 PMC2430252

[6] Cisse RS, Hamburg JD, Freeman ME, Davis AJ. Using locally produced millet as a feed ingredient for poultry production in Sub-Saharan Africa. Journal of Applied Poultry Research. 2017; 26:9–22.

[7] Qaisrani SN, Murtaza S, Khan AH, Bibi F, Iqbal SMJ, Azam F, et al. Variability in millet: Factors influencing its nutritional profile and zootechnical performance in poultry. Journal of Applied Poultry Research. 2019; 28:242–252.

[8] Liu SY, Selle PH, Cowieson AJ. Strategies to enhance the performance of pigs and poultry on sorghum-based diets. Animal Feed Science and Technology. 2013; 181:1–14.

[9] Manyelo TG, Ng’ambi JW, Norris D, Mabelebele M. Substitution of *Zea mays* by *Sorghum bicolor* on performance and gut histo-morphology of Ross 308 broiler chickens aged 1–42 d. Journal of Applied Poultry Research. 2019; 28:647–657.

[10] Liu SY, Fox G, Khoddami A, Neilson KA, Truong HH, Moss AF, et al. Grain sorghum: A conundrum for chicken-meat production. Agriculture. 2015; 5:1224–1251.

[11] Saleh ASM, Zhang Q, Chen J, Shen Q. Millet grains: Nutritional quality, processing, and potential health benefits. Comprehensive Reviews in Food Science and Food Safety. 2013; 12:281–295.

[12] Selle PH, Truong HH, Khoddami A, Moss AF, Roberts TH, Liu SY. The impacts of hammer-mill screen size and grain particle size on the performance of broiler chickens offered diets based on two red sorghum varieties. British Poultry Science. 2019; 60:209–218. 10.1080/00071668.2016.1257777 27848267

[13] Behnke KC. Feed manufacturing technology: current issues and challenges. Animal Feed Science and Technology. 1996; 62:49–57.

[14] National Research Council. Nutrient Requirements of Poultry. 9th rev. ed. NRC, National Academy Press, Washington, DC, USA. 1994.

[15] AOAC. Official Methods of Analysis of Association of Official Analytical Chemists International, 16th ed. Association of Official Analytical Chemists, Arlington, VA, USA. 2005.

[16] Livesey G. Procedure for calculating the digestible and metabolisable energy values of food components making a small contribution to dietary intake. Journal of the Science of Food and Agriculture. 1989; 48:475–481.

[17] Washington IM, van Hoosier G. Clinical biochemistry and hematology. University of Washington, Seattle, WA, USA. 2012. p. 59–91.

[18] CIE. Recommendations on uniform color spaces-color difference equations, Psychometric Colour Terms. Supplement No. 2 to CIE Publication No. 15 (E-1.3.1) 1978, 1971/(TC-1-3). Commission Internationale de l’Eclairage, Paris, France. 1976.

[19] Grau R, Hamm R. About the water-binding capacity of the mammalian muscle. II. Communication. Zeitschrif Lebensm Unters Briskly. 1957; 105:446.

[20] Zhang X, Gao T, Song L, Zhang L, Jiang Y, Li JL, et al. Effects of different thawing methods on the quality of chicken breast. International Journal of Food Science and Technology. 2017; 52:2097–2105.

[21] Honikel KO. Reference methods for the assessment of physical characteristics of meat. Meat Science. 1998; 49:447–570. 10.1016/s0309-1740(98)00034-5 22060626

[22] SAS. Users Guide: Statistics. Version 9.3. Cary: SAS Institute, NC, USA. 2010.

[23] Ahmed MA, Dous BM, Atti KAA. Effect of substituting yellow maize for sorghum on broiler performance. Journal of World’s Poultry Research. 2013; 3:13–17.

[24] Leandro NSM, Stringuini JH, Café MB, Orsine GF, Rocha AC. Effect of granulometry of corn and soybean meal on the performance of Japanese quails. Brazilian Journal of Animal Science. 2001; 30:1266–1271.

[25] Gualtieri M, Rapaccini S. Sorghum grain in poultry feeding. World’s Poultry Science Journal. 1990; 46:246–254.

[26] Taylor JRN, Emmambux MN. Developments in our understanding of sorghum polysaccharides and their health benefits. Cereal Chemistry. 2010; 87:263–271.

[27] Beta T, Corke H. Effect of ferulic acid and catechin on sorghum and maize starch pasting properties. Cereal Chemistry. 2004; 81:418–422.

[28] Oatway L, Vasanthan T, Helm JH. Phytic acid. Food Reviews International. 2001; 17: 419–431.

[29] Selle PH, Walker AR, Bryden WL. Total and phytate-phosphorus contents and phytase activity of Australian-sourced feed ingredients for pigs and poultry. Australian Journal of Experimental Agriculture. 2003; 43:475–479.

[30] Truong HH, Liu SY, Selle PH. Starch utilization in chicken-meat production: The foremost influential factors. Animal Production Science. 2015; 56:797–814.

[31] Kwari ID, Igwebuike JU, Mohammed ID, Diarra SS. Growth, hematology and serum chemistry of broiler chickens fed raw or processed Sorrel (*Hibiscus sabdariffa*) seed meal in a semi-arid environment. International Journal of Natural Sciences. 2011; 2:22–27.

[32] Kwari ID, Diarra SS, Saleh B, Bovoa PR, Ramat OA, Tochukwu D. Growth, hematology and serology of broiler chickens fed different cultivars of sorghum as replacement for maize in the semi-arid zone of Nigeria. International Journal of Poultry Science. 2011; 10: 608–612.

[33] Tandiang DM, Diop MT, Dieng A, Yoda GML, Cisse N, Nassim M. Effect of corn substitution by sorghum grain with low tannin content on broilers production: Animal performance, nutrient digestibility and carcass characteristics. International Journal of Poultry Science. 2014; 13:568–574.

[34] Xu Y, Stark CR, Ferket PR, Williams CM, Brake J. Effects of feed form and dietary coarse ground corn on broiler live performance, body weight uniformity, relative gizzard weight, excreta nitrogen, and particle size preference behaviors. Poultry Science. 2015; 94:1549–1556. 10.3382/ps/pev074 25881586

[35] Issa S, Hancock JD, Tuinstra MR, Kapran I, Kaka S. Effects of sorghum variety on growth and carcass characteristics in broiler chicks reared in West Africa. Poultry Science. 2007; 86:59–69. 10.1093/ps/86.1.59 17179416

[36] Raju MVLN, Shyam SG, Sadagopan VR, Elangovan AV, Reddy MR, Rama Rao SV. Replacement of maize with jowar, bajra or ragi in broiler chicken diets. Animal Nutrition Feed Technology Journal. 2004; 4:53–61.

[37] Rama Rao SV, Raju MVLN, Panda AK. Replacement of yellow maize with graded levels of pearl millet (*Pennisetum typhoides*) in commercial broiler diets. Indian Journal of Poultry Science. 2003; 38:236–242.

[38] Muchenje V, Dzama K, Chimonyo M, Strydom PE, Raats JG. Relationship between pre-slaughter stress responsiveness and beef quality in three cattle breeds. Meat Science. 2009; 81:653–657. 10.1016/j.meatsci.2008.11.004 20416575

[39] Barbut S. Colour measurements for evaluating the pale soft exudative (PSE) occurrence in turkey meat. Food Research International. 1993; 26:39–43.

[40] Węglarz A. Meat quality defined based on pH and color depending on cattle category and slaughter season. Czech Journal of Animal Science. 2010; 55:548–556.

[41] Northcutt JK, Foegeding EA, Edens FW. Water-holding properties of thermally preconditioned chicken breast and leg meat. Poultry Science. 1994; 73:308–316. 10.3382/ps.0730308 8146078

[42] Mancini RA, Hunt MC. Current research in meat color. Meat Science. 2005; 71:100–121. 10.1016/j.meatsci.2005.03.003 22064056

