# Supplementary material for: Complete replacement of maize grain with sorghum and pearl millet grains in Jumbo quail diets: Feed intake, physiological parameters, and meat quality traits
Source: PLoS One. 2021 Mar 29;16(3):e0249371. doi: 10.1371/journal.pone.0249371 (PMC8007063; doi:10.1371/journal.pone.0249371)
Supplement: S1 Table — (DOCX) [file pone.0249371.s001.docx]

**S1 Table. Average weekly feed intake (g/bird) in Jumbo quail reared on whole or crushed sorghum and pearl millet grains-based diets**

|  | **^1^Diets** | | | | |  |  |
| --- | --- | --- | --- | --- | --- | --- | --- |
|  | **CON** | **WSG** | **CSG** | **WMG** | **CMG** | **^2^SEM** | ***P* value** |
| Week 3 | 554.3 | 556.9 | 548.8 | 553.2 | 544.7 | 6.49 | 0.630 |
| Week 4 | 653.5 | 655.9 | 646.0 | 654.5 | 646.7 | 3.38 | 0.086 |
| Week 5 | 883.3 | 882.4 | 877.0 | 869.0 | 818.3 | 22.22 | 0.162 |
| Week 6 | 971.1 | 977.5 | 1021.5 | 1002.2 | 931.1 | 28.28 | 0.160 |

^1^Diets: CON = maize grain-based commercial grower diet; WSG = commercial grower diet in which maize grain was replaced with whole sorghum grain; CSG = commercial grower diet in which maize grain was replaced with crushed sorghum grain; WMG = commercial grower diet in which maize grain was replaced with whole millet grain; CMG = commercial grower diet in which maize grain was replaced with crushed millet grain.

^2^SEM = standard error of the mean.
