# Supplementary material for: Complete replacement of maize grain with sorghum and pearl millet grains in Jumbo quail diets: Feed intake, physiological parameters, and meat quality traits
Source: PLoS One. 2021 Mar 29;16(3):e0249371. doi: 10.1371/journal.pone.0249371 (PMC8007063; doi:10.1371/journal.pone.0249371)
Supplement: S2 Table — (DOCX) [file pone.0249371.s002.docx]

**S2 Table. Average weekly body weight (g/bird) in Jumbo quail reared on whole or crushed sorghum and pearl millet grains-based diets**

|  | **^1^Diets** | | | | |  |  |
| --- | --- | --- | --- | --- | --- | --- | --- |
|  | **CON** | **WSG** | **CSG** | **WMG** | **CMG** | **^2^SEM** | ***P* value** |
| Initial weight | 72.97 | 75.30 | 72.47 | 77.26 | 76.64 | 3.791 | 0.826 |
| Week 3 | 128.6^c^ | 127.6^bc^ | 113.1^ab^ | 134.0^c^ | 109.3^a^ | 3.994 | 0.0001 |
| Week 4 | 179.8^b^ | 176.2^b^ | 153.1^a^ | 183.8^b^ | 131.7^a^ | 5.574 | <.0001 |
| Week 5 | 208.8^b^ | 215.5^b^ | 192.5^b^ | 219.0^b^ | 140.6^a^ | 7.318 | <.0001 |
| Week 6 | 225.0^b^ | 234.1^b^ | 215.3^ab^ | 237.7^b^ | 190.8^a^ | 11.05 | 0.024 |

^a,b,c^ In row, means with common superscripts do not differ (*P* > 0.05).

^1^Diets: CON = maize grain-based commercial grower diet; WSG = commercial grower diet in which maize grain was replaced with whole sorghum grain; CSG = commercial grower diet in which maize grain was replaced with crushed sorghum grain; WMG = commercial grower diet in which maize grain was replaced with whole millet grain; CMG = commercial grower diet in which maize grain was replaced with crushed millet grain.

^2^SEM = standard error of the mean.
